# Systemic *Escherichia coli* infection does not influence clinical symptoms and neurodegeneration in experimental autoimmune encephalomyelitis

**DOI:** 10.1186/s12868-015-0172-4

**Published:** 2015-06-19

**Authors:** Prateek Kumar, Katharina Friebe, Rieka Schallhorn, Zahra Moinfar, Roland Nau, Mathias Bähr, Sandra Schütze, Katharina Hein

**Affiliations:** Department of Neurology, University Hospital, University Medical Center Göttingen, Robert-Koch-Strasse 40, 37075 Göttingen, Germany; Institute of Neuropathology, University Medical Center Göttingen, 37075 Göttingen, Germany

**Keywords:** Multiple sclerosis, Experimental autoimmune encephalomyelitis, Infection, *E. coli* neurodegeneration

## Abstract

**Background:**

Systemic infections can influence the course of multiple sclerosis (MS), especially by driving recurrent acute episodes. The question whether the infection enhances tissue damage is of great clinical importance and cannot easily be assessed in clinical trials. Here, we investigated the effects of a systemic infection with *Escherichia coli*, a Gram-negative bacterium frequently causing urinary tract infections, on the clinical course as well as on neurodegeneration in experimental autoimmune encephalomyelitis (EAE), an animal model of MS.

**Methods:**

Rats were immunized with myelin oligodendrocyte glycoprotein (MOG_1–125_) and challenged intraperitoneally with live *E. coli* K1 in the preclinical or in the clinical phase of the disease. To ensure the survival of animals, antibiotic treatment with ceftriaxone was initiated 36 h after the infection and continued for 3 consecutive days.

**Results:**

Systemic infection with *E. coli* did not influence the onset of clinical EAE symptoms or disease severity. Analysis of the optic nerve and retinal ganglion cells revealed no significant changes in the extent of inflammatory infiltrates, demyelination and neurodegeneration after *E. coli* infection.

**Conclusions:**

We could not confirm the detrimental effect of lipopolysaccharide-induced systemic inflammation, a model frequently used to mimic the bacterial infection, previously observed in animal models of MS. Our results indicate that the effect of an acute *E. coli* infection on the course of MS is less pronounced than suspected and underline the need for adequate models to test the role of systemic infections in the pathogenesis of MS.

## Background

Multiple sclerosis (MS) is the most frequent inflammatory disease of the central nervous system that often manifests with acute optic neuritis. The cause of MS is not known, but several factors have been shown to be associated with the risk of developing this disease including vitamins [1], smoking [2], genetic factors [3, 4], and infections [5]. A link between the first clinical attack or relapses in MS and infections has been proposed on the basis of epidemiological studies [6]. There is a growing body of evidence that the onset and progression of MS is influenced by systemic bacterial infections. In MS patients, resolution of neurological symptoms that occurred during an infection is often incomplete [7, 8]. Whether this phenomenon is a consequence of truly aggravated neurodegeneration during the systemic infection is a question of great clinical importance.

In the majority of experimental studies in this area, lipopolysaccharide (LPS), a cell wall component of Gram-negative bacteria with strong immunostimulatory properties, has been used to induce systemic inflammation. Systemic treatment of animals with LPS has been shown to promote relapses [9] and to enhance neurodegeneration in experimental models of MS [10]. Although frequently used for this purpose in animal models, LPS-induced inflammation does not accurately mimic a systemic infection with viable bacteria [11, 12]. Only few studies in humans or animal models demonstrated an exacerbating effect of an acute infection with Gram-negative bacteria on the disease course of MS and EAE [13–15]. Here, the upper respiratory tract pathogen *Clamydia pneumoniae* is the most thoroughly investigated candidate. However, a large number of MS patients suffers from urinary bladder dysfunction, which in turn leads to urinary tract infections (UTIs) [16] and results in significant morbidity and impairment in quality of life. *Escherichia coli* (*E. coli*) is a Gram-negative bacterium and the most common cause of UTI in MS patients. In the present study, we investigated the impact of a systemic *E. coli* infection during the preclinical and clinical phase of experimental autoimmune encephalomyelitis (EAE), an animal model of MS induced by immunization with myelin oligodendrocyte glycoprotein (MOG) in female Brown Norway rats, which shows a progressive disease course without any definite form of remission. We have previously demonstrated that this model strongly reflects the neurodegenerative aspects of MS. 12–14 days after immunization with MOG, more than 90% of affected animals develop acute optic neuritis, which leads to acute axonal degeneration of the optic nerve and consecutive apoptosis of retinal ganglion cells (RGCs) [17–19]. In contrast, the amount of spinal cord lesions shows certain variability in this model [20–22].

## Methods

### Rats

Female Brown Norway rats, 8–10 weeks of age, were used in all experiments. Animals were obtained from Charles River (Sulzfeld; Germany) and kept under environmentally controlled and pathogen-free conditions. All experiments involving animal use were performed in accordance with the relevant laws and institutional guidelines. All animal experiments were approved by the Animal Care Committee of the University Hospital of Göttingen, Germany, and by the Niedersächsisches Landesamt für Verbraucherschutz und Lebensmittelsicherheit (LAVES), Braunschweig, Lower Saxony; Germany.

### Retrograde labelling of RGCs

For retrograde labelling of retinal ganglion cells, rats were anesthetized by intraperitoneal injection of ketamine (Ketanest 10; 0.95 ml/kg; Atarost, Twistringen; Germany) and xylazine 2% (0.25 ml/kg; Albrecht, Aulendorf; Germany) and positioned in a stereotaxic frame. The skin was incised mediosagittaly, and holes were drilled into the skull above each superior colliculus (6.8 mm dorsal and 2 mm lateral from bregma). 2 µl of the fluorescent dye Fluoro-Gold (5% in distilled water; Fluorochrome, Englewood, CO, USA) were injected into both superior colliculi using a 10 µl Hamilton syringe.

### Induction of EAE and infection with *E. coli*

**Figure 1:**
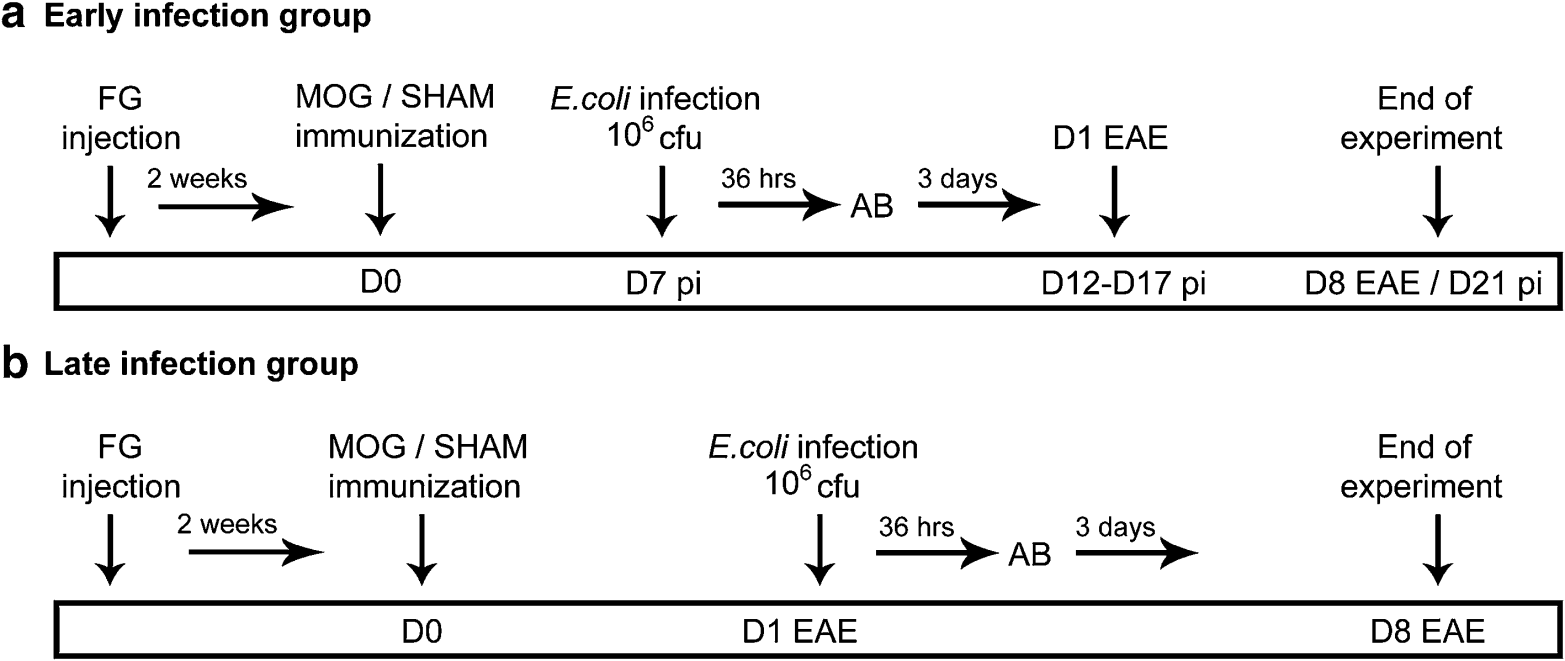
Experimental design. Fluoro-Gold (FG) injection was performed 2 weeks prior to MOG immunization. **a** In the early infection group, intraperitoneal injection with *E. coli* or saline was performed on day (*D*) seven post immunization followed by antibiotic treatment (*AB*) for 3 consecutive days. Day 12–17 was the usual time for clinical onset of the disease. Animals were followed until day 8 of EAE or day 21 post immunization in case of clinically healthy animals. **b** In the late infection group intraperitoneal injection with *E. coli* or saline was performed in animals on the day of clinical manifestation of EAE. Animals were followed until day 8 of EAE. Tissue was harvested from the optic nerve for histopathological analysis, retinas were flat-mounted for quantification of retinal ganglion cells.

Rats were anesthetized by inhalation anaesthesia with isoflurane and injected subcutaneously at the base of the tail with a total volume of 200 µl inoculum, containing 50 µg recombinant rat MOG^Igd^ (Kindly provided by C. Stadelmann, Department of Neuropathology, Göttingen; Germany) in saline emulsified (1:1) with complete Freund’s adjuvant (CFA; Sigma-Aldrich, St. Louis, MO, USA) containing 200 µg heat-inactivated *Mycobacterium tuberculosis* (strain H 37 RA; Difco Laboratories, Detroit, MI, USA). Immunized animals were randomly assigned into four different groups. The animals were infected intraperitoneally with 10^6^ colony-forming units (CFU) *E. coli* K1 (originally isolated from the cerebrospinal fluid of a child with meningitis; gift G. Zysk, Düsseldorf; Germany) in 400 µl 0.9% saline (B. Braun, Melsungen; Germany) either on day 7 post immunization (“early infection” group; Figure 1a) or on day one of clinical manifestation of EAE (“late infection” group; Figure 1b). Control rats received an intraperitoneal injection of an equal volume of saline.

The infection time point 7 days after immunization was chosen to elucidate whether an infection during the asymptomatic phase could modulate the time point of disease onset in MOG-EAE. The number of animals in this experiment used for analysis was 28 (n = 15, “early infection”; n = 13, control group) excluding the animals which died after *E. coli* infection or were sacrificed for ethical reasons. To mimic the clinical situation in MS patients, animals were infected on day one of EAE (“late infection” group). Here, we addressed the question whether the infection aggravates the clinical symptoms of EAE when it occurs in the clinical phase of the disease. The number of animals included in this experiment used for analysis was 29 (n = 15, “late infection”; n = 14, control group). Data of animals that died as a consequence of the *E. coli* infection were not included in the analysis.

To ensure the survival of *E. coli*-infected animals, antibiotic treatment was started 36 h after the infection in both groups. All animals received 100 mg/kg ceftriaxone (Hikma pharma; Klein-Winternheim; Germany) twice daily for 3 consecutive days. To confirm the systemic infection in the “late infection” group, blood samples were collected from the tail vein under inhalation anaesthesia with isoflurane 24 h after the infection (n = 8 in each group). Bacterial concentrations in blood were determined by quantitative plating of blood samples on blood agar plates followed by incubation for 24 h at 37°C. The bacterial detection limit was 100 colony forming units (CFU)/ml. Animals were followed until day 8 after the clinical manifestation of the disease (EAE d8) in the “early infection” and the “late infection” experiment or until day 21 post immunization in case of clinically healthy animals in the “early infection” experiment. The rationale for the study end point at day 8 of EAE lies in our previous studies in this animal model showing a gradual neurodegeneration with significant reduction of RGCs until day 8 of EAE. No further substantial changes neither in RGCs density nor in the histopathological changes of the optic nerve were observed beyond this time point when compared to day 21 of EAE [17, 18].

### Clinical evaluation

Rats were scored for clinical signs of EAE and weighed daily. Clinical signs were scored as follows: grade 0, no symptoms; grade 0.5, distal paresis of the tail; grade 1, complete tail paralysis; grade 1.5, paresis of the tail and mild hind limb paresis; grade 2.0, unilateral severe hind limb paresis; grade 2.5, bilateral severe hind limb paresis; grade 3.0, complete bilateral hind limb paralysis; grade 3.5, complete bilateral hind limb paralysis and paresis of one front limb; grade 4, complete paralysis (tetraplegia), moribund state, or death.

### Histopathology and immunohistochemistry

Histopathological evaluations were performed on paraformaldehyde-fixed, paraffin-embedded 2 µm-thick slices of the optic nerves (ONs). Luxol Fast Blue staining was performed to assess demyelination. Photographs of vertical cross sections were taken using an AxioCam MR Microscopy camera (Zeiss, Göttingen; Germany). The images were processed using Zeiss AxioVision 4.2 software to evaluate the demyelinated area as a percentage of the whole optic nerve cross-section. Immunohistochemistry was performed on paraffin-embedded ON cross-sections representing three different levels of an optic nerve. ED1 macrophages/activated microglia (MCA341R diluted 1:500; Serotec, Oxford; UK) were detected in cross-sections using avidin–biotin detection. Spleen sections served as positive control for ED1 staining. ED1-positive cells were evaluated using the following score: 0, no labelled cells; 1, a few ED1-positive cells (number countable, infiltration of less than 10% of the ON cross-section area); 2, infiltration of less than 10% of the ON cross-section area with ED1 positive cells in at least one of the three cross-sections of an ON, number of ED1-positive cells not countable; 3, infiltration of 10–50% of the ON cross-section area with labelled ED1-positive cells in at least in one of three cross-sections of an ON; 4, infiltration of 50–80% area of the ON cross-section area in at least one of the three cross-sections of an ON; 5, infiltration of more than 80% of the ON cross-section area in at least one of the three cross-sections of an ON. Bielschowsky’s silver impregnation was performed on 2 µm-thick cross-section slices of the ON to access the axonal density on the ON cross-sections. The density of axons in each ON was measured semi-quantitatively in at least nine standardized microscopic fields for each ON by using a 25 points grid system. Mean axon density was calculated for each ON.

### Quantification of RGC density

At day 8 of MOG-EAE, retinas were dissected, flat-mounted on glass slides, and examined by fluorescence microscopy (Zeiss Axioplan 2) using a DAPI filter (315/395 nm). RGC densities were determined by counting labelled cells in three areas per retinal quadrant at three different eccentricities of 1/6, 3/6, and 5/6 of the retinal radius. Cell counts were performed by two independent investigators following a blinded protocol.

### Statistics

Data presented here are the cumulative results of three independent experiments and presented as mean ± SEM. Student’s *t* test (parametric analysis) was used to compare RGC densities, demyelination and axonal densities within the ONs between two groups. For the comparison of bacterial concentrations, ED1 scores, and mean clinical scores of EAE between two groups (nonparametric analysis), the Mann–Whitney U-test was used. A *P* value (*P*) ≤0.05 was considered statistically significant.

## Results

### Intraperitoneal injection of *E. coli* leads to systemic infection with weight loss or bacteraemia

**Figure 2:**
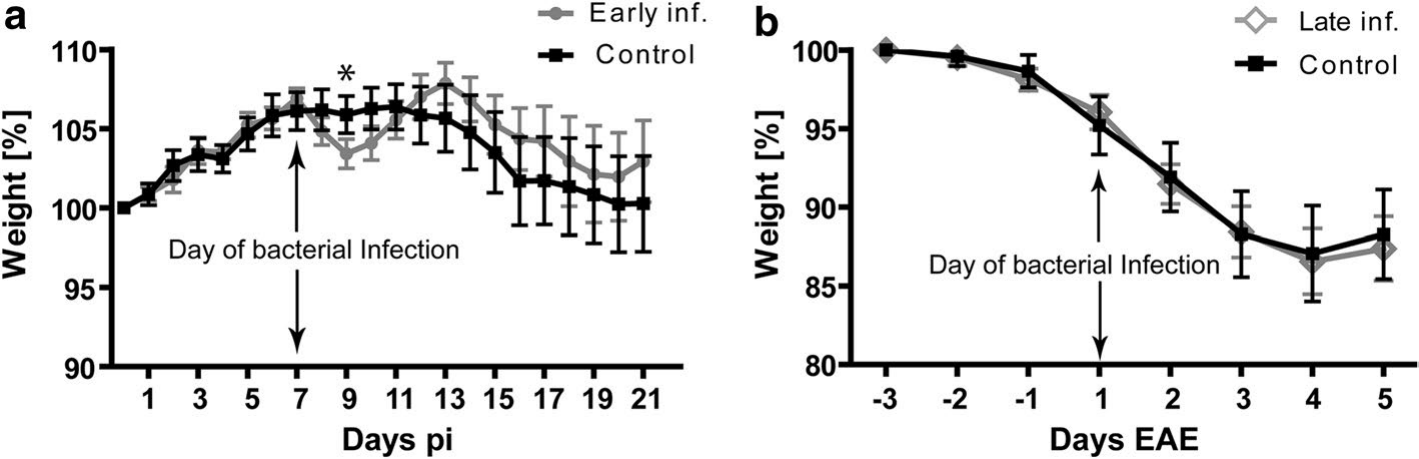
Comparison of weight loss during the course of the experiments; **a** represent the weight curve in the animals infected on day 7 post immunization along with their control (early infection group). **b** represent the weight curve of the group of animals, when infection was performed on the day of clinical onset of the disease (late infection group) with their respective control.

*E. coli* infection was either performed on day 7 post immunization (“early infection”) or on day 1 of clinical EAE (“late infection”). In the “early infection” experiment, animals infected with *E. coli* showed a significant weight loss after the infection whereas animals in the control group did not lose weight (*P* < 0.001; Figure 2a). Infected animals recovered their weight after the antibiotic treatment had been started. In contrast, in the “late infection” experiment, animals infected with *E. coli* did not show any significant difference in weight loss compared to the control group (*P* = 0.69; Figure 2b). This is probably due to the fact that during the clinical disease course of EAE both infected and control groups lost their weight rapidly. To confirm the systemic infection, *E. coli* concentrations were determined in blood samples collected 24 h after the infection. Animals infected with *E. coli* showed high bacterial counts in blood (Log_10_ 3.64 CFU/ml ± 3.32), whereas no bacteria were detected in blood of control animals, which received saline instead of the bacterial inoculum. The overall animal mortality after *E. coli* infection was around 20%.

### *E. coli* infection has no impact on onset and severity of clinical symptoms in MOG-EAE

**Figure 3:**
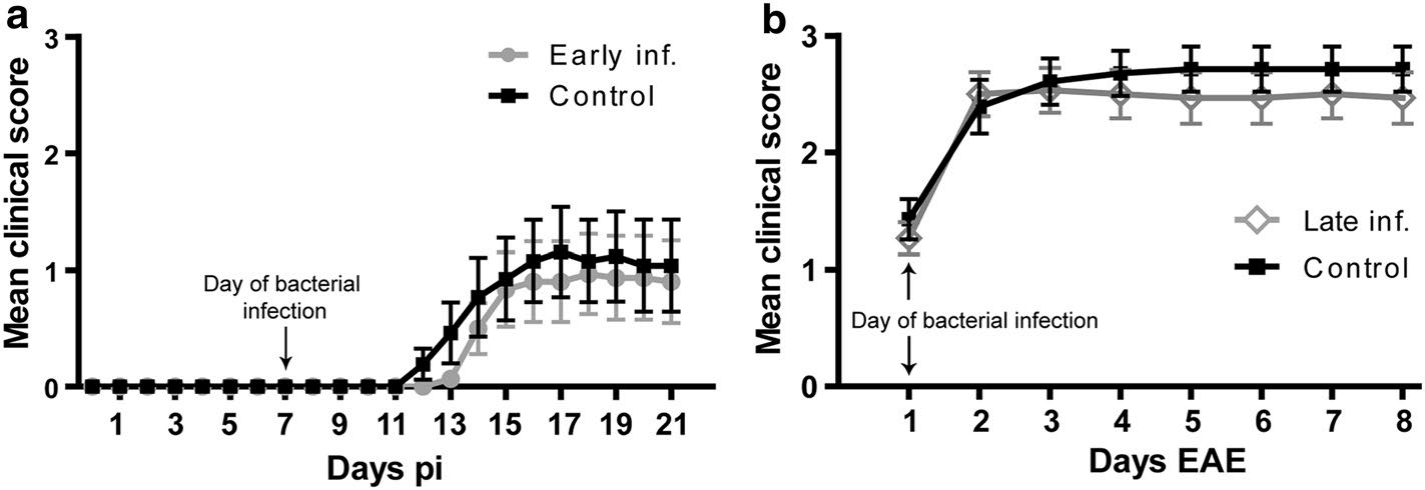
Comparison of clinical course of the disease: the mean clinical score represents the neurological deficit based on spinal cord lesion. **a** represent the clinical course of the disease in the animals infected on day 7 post immunization (early infection group). **b** represent the clinical course of the disease when infection was performed on the day of clinical onset of the disease (late infection group). Data are shown as mean ± SEM of the daily score.

In the “early infection” experiment, 7 out of 15 *E. coli* infected animals showed clinical disease symptoms related to the spinal cord lesions, which did not differ much from the control, where 7 out of 13 animals developed clinical score of EAE. In contrast, the incidence of optic neuritis in “early infection” experiment was 87% in animals infected with *E. coli* and 92% in control group. The mean clinical score of infected animals and control animals, at the end of the experiments was not significantly different [0.9 ± 0.35 (n = 15) vs. 1.04 ± 0.39 (n = 13); *P* = 0.8; Figure 3a]. Also the mean time point of clinical onset of EAE was similar in both groups (14.17 ± 0.48 days post immunization in the infection group and 13.50 ± 0.62 days post immunization in the control group; *P* = 0.41).

In the “late infection” experiment, infection was induced only in animals, which showed clinical sign of EAE. The incidence of optic neuritis was 100% in both the animal groups. In this experiment, we observed that *E. coli* infection performed on day 1 of clinical onset of the disease also did not substantially modulate the course of EAE. Here, the mean clinical score at the end of the experiment was not significantly different between the group infected with *E. coli* and the respective control group [2.47 ± 0.22 (n = 15) vs. 2.71 ± 0.19 (n = 14); *P* = 0.51; Figure 3b].

### *E. coli* infection does not influence demyelination, inflammatory infiltration and axonal density of the optic nerve

**Figure 4:**
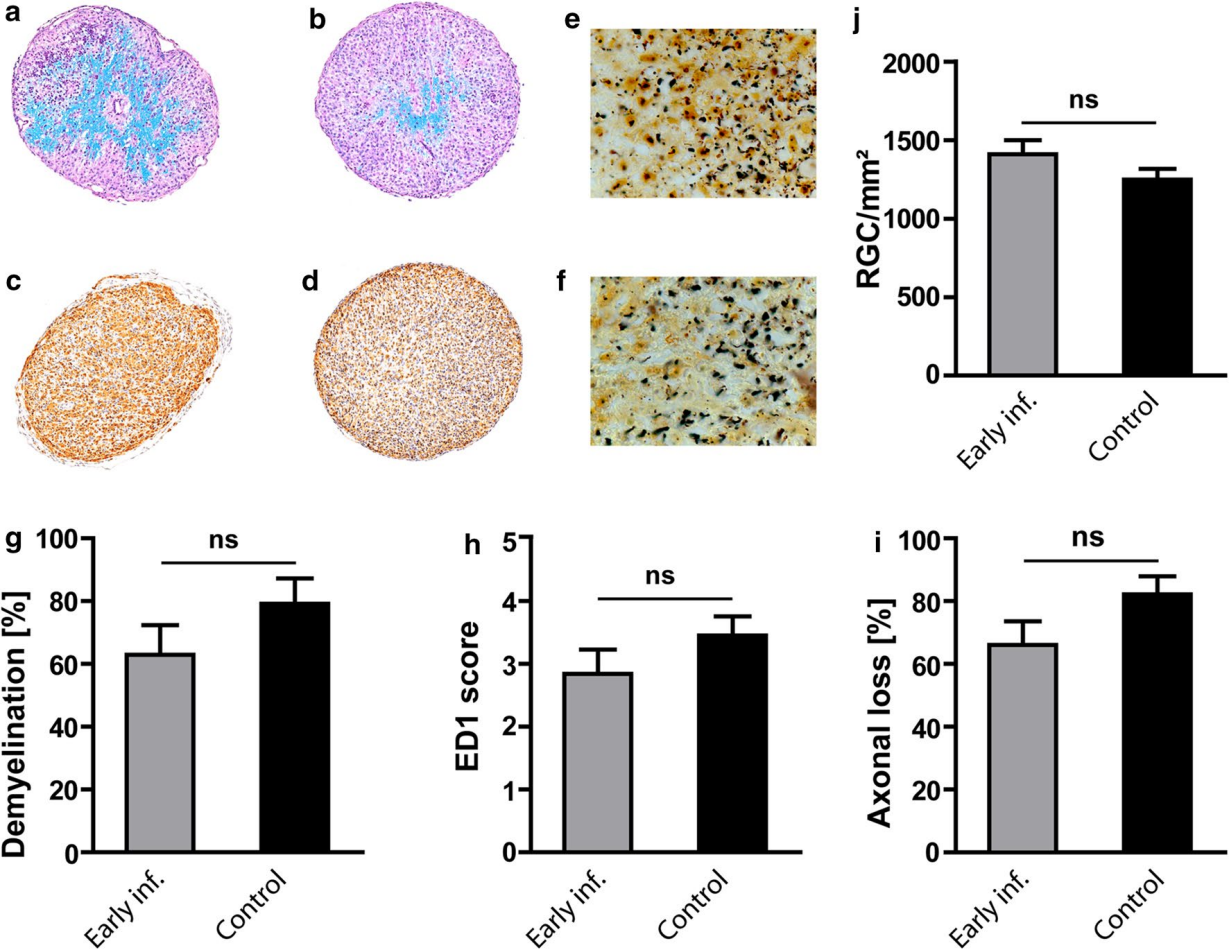
Optical nerve (ON) histopathology for the early infection group. **a**, **b** Representative Luxol Fast Blue-stained cross sections of ONs of an animal infected with *E. coli* (**a**) and an animal from the respective control group (**b**). Representative examples for the number of infiltrated ED1 macrophages/activated microglia detected in the ON of an *E. coli* infected animal (**c**) and an animal from the non-infected control group (**d**). **e**, **f** Bielschowsky’s silver impregnation of an ON cross-section revealed no differences in axonal densities in a MOG immunized animal infected on day 7 post immunization (**e**) compared to a non-infected respective control animal (**f**). **j** Represents the quantitative data for numbers of surviving retinal ganglion cells (RGCs) of the animals infected with *E. coli* and of the animals without infection. **g**, **h** and **i** represent the quantitative data for demyelination, inflammatory infiltrates and for axonal counts in both the *E. coli* infected and control group (n = 15 infected; n = 13 control). *Bar length* for Luxol Fast Blue, ED1, 100 µm, and for Bielschowsky’s silver impregnation = 20 µm.

In optic nerves evaluated histopathologically at the end of the experiment, the extent of demyelination was similar in animals infected with *E. coli* on day 7 post immunization and the respective control animals [62.90 ± 9.43% (n = 15) vs. 79.18 ± 8.06% (n = 13); *P* = 0.20; Figure 4a, b, g]. Analysis of inflammatory infiltrates by ED1 staining also revealed no significant difference between the infection group and the control group in the “early infection” experiment [2.84 ± 0.39 (n = 15) vs. 3.45 ± 0.3 (n = 13) in the control group; *P* = 0.50; Figure 4c, d, h]. Bielschowsky silver impregnation was performed to assess the axonal density of the ON. The axonal loss in infected animals and control animals did not differ significantly [66 ± 7.64% (n = 15) vs. 82.19 ± 5.69% (n = 13); *P* = 0.10; Figure 4e, f, i].

**Figure 5:**
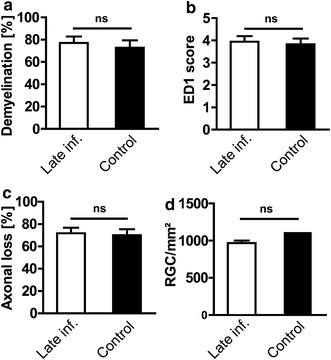
Optic nerve (ON) histopathology in the late infection group. **a**, **b**, and **c** represents the quantitative data for demyelination, inflammatory infiltrates (ED1 score), and axonal counts in the optic nerve from the animals infected on the day of clinical manifestation of EAE and the animals from respective control group. **d** Represents the quantitative data for numbers of surviving retinal ganglion cells (RGCs) of the animals infected with *E. coli* on day 1of EAE and of the animals without infection.

In the “late infection” experiment, we also did not find any effect of *E. coli* infection on inflammatory infiltration [3.94 ± 0.24 (n = 15) in the infection group vs. 3.83 ± 0.26 (n = 14) in the control group; *P* = 0.74; Figure 5b] or demyelination [77.06 ± 5.77% (n = 15) in the infection group vs. 72.87 ± 6.46% (n = 14) in the control group; *P* = 0.63; Figure 5a] of the optic nerve at the end of the experiment. Moreover, we did not find any difference in axonal density. The extent of axonal loss was 71.84 ± 5.03% (n = 15) in the infection group and 70.07 ± 5.39% (n = 14) in the control group (*P* = 0.81; Figure 5c).

### *E. coli* infection does not affect neuronal viability

To evaluate the impact of *E. coli* infection on neuronal damage in MOG-EAE, numbers of surviving RGCs were counted on Fluoro-Gold-labeled retinal flat mounts. In accordance with the histopathological changes in the ON, analysis of RGC density did not reveal any significant differences between infected animals and the respective control animals both in the “early infection” experiment [1,411 ± 88 vs. 1,248 ± 69/mm^2^; *P* = 0.15; Figure 4j, (n = 25 each)] and the “late infection” experiment [967 ± 33 vs. 1,116 ± 78/mm^2^; *P* = 0.08; Figure 5d, (n = 16 each)]. For RGC counts ‘‘n’’ indicate the number of eyes used for quantification. RGC counts in both experiments were significantly lower than those of healthy control animals in our previous studies (2,567 ± 89; mean ± SEM) [23], indicating a loss of RGCs in infected and non-infected animals.

## Discussion and conclusions

There is a growing body of evidence that the onset and progression of MS is influenced by systemic infections caused by bacteria or viruses. Bacterial infections can aggravate neurological symptoms, which remain even after the infection is resolved, while some appears to be protective [24]. A better understanding of the mechanisms underlying these phenomena would be an essential step for a rational treatment of infections in MS patients. In spite of the rising interest in the field linking infection and MS, only few studies examined the impact of real systemic infections with viable bacteria in animal models of MS. In the present study, we investigated whether systemic infection with *E. coli*, a Gram-negative bacterium frequently causing urinary tract infection, has a disease-modulating effect on EAE.

Clinical as well as experimental data indicate the preclinical phase of MS to be an “at risk” period with the greatest susceptibility to infections [25, 26]. Systemic infection with *Streptococcus pneumoniae* 7 days after immunization with MOG aggravated EAE symptoms in mice, whereas the severity of EAE symptoms was unaltered in mice infected 2 days before or 21 days after MOG-immunization [27]. For this reason, in our study, animals were infected at day 7 post immunization corresponding to the induction phase of EAE. Intraperitoneal injection of *E. coli* induced weight loss in EAE animals indicating severe systemic infection and sickness behavior in animals. However, systemic *E. coli* infection during the latent phase of EAE did not influence the time point of onset of clinical symptoms or the disease severity of EAE in this model. To mimic the situation in MS patients at the beginning of a clinical event, animals were infected on the first day of onset of EAE symptoms. Despite the presence of high bacterial counts in the systemic circulation of infected animals, the severity of EAE symptoms was not influenced by *E. coli* infection.

It is poorly understood whether systemic infections lead to tissue damage in the CNS with increased neurodegeneration, a major histopathological correlate of disability in MS patients. The results of magnetic resonance imaging (MRI) studies on the influence of extra cerebral infections on the number of contrast-enhancing MS lesions as a marker of disease activity are inconsistent [28, 29]. Moreover, reliable markers for neurodegeneration in MS patients do not exist. For this reason, much of the knowledge about the interaction of systemic infection and autoimmunity in MS is derived from studies in EAE models. Previous histopathological analyses revealed that a single dose of LPS increased the inflammatory response and demyelination in EAE lesions [30]. In addition, it led to a switch in the macrophage/microglia phenotype towards proinflammation increasing axonal damage in an EAE model [10]. In contrast, our histopathological analysis performed on cross-sections of the optic nerve did not reveal any significant increase in inflammatory infiltrates in the animals infected with *E. coli* either on day 7 post immunization or on day 1 of EAE. In our study, we also did not observe any significant differences in other histopathological hallmarks such as demyelination and axonal damage between animals infected with *E. coli* and the respective control animals. In accordance with the histopathology of the optic nerve, the survival of RGCs was also not significantly influenced by the *E. coli* infection.

Given the disease-modulating effect of systemic LPS in EAE [9, 10] our data are surprising. In accordance with our observations, the disease-aggravating effect of LPS observed in other neurodegenerative disease models [31, 32] could also not be confirmed in mouse models of Alzheimer’s and Parkinson’s disease when real systemic infections were induced by viable *S. pneumoniae* [31, 33] or *E. coli* (Schütze et al., unpublished observation).

There are several hypotheses that may explain the discrepancy between the effect of LPS-induced systemic inflammation and systemic *E. coli* infection on clinical symptoms and neurodegeneration in EAE. The LPS doses applied in the studies mentioned above were very high in comparison to plasma endotoxin levels measured in patients with sepsis [34]. Consequently, the levels of the released pro-inflammatory cytokines (e.g. TNF-alpha, IL-1) in sepsis patients are much lower than those typically found after endotoxin infusion [35–37]. In-vitro data suggest that microglial activation and microglia-mediated neurodegeneration depend on the dose of the inflammatory stimulus [38, 39]. We assume that stimulation of the innate immune response in the CNS induced by the systemic *E. coli* infection in our study was less severe than that achieved by high doses of LPS and did not reach a sufficient level to induce neurodegeneration.

Treatment with the β-lactam antibiotic ceftriaxone was initiated 36 h after the infection in our experiments which stops bacterial application and terminates the effect of infection. Ceftriaxone has a bacteriolytic mode of action, thereby causing the rapid release of high amounts of bacterial products acting as strong stimuli of innate immunity [40]. Therefore, it is tempting to assume that an antibiotic treatment of infection may have an at least temporary effect on the course of EAE. The fact that the control group reached more severe disease scores in both the experiments argues against an aggravation of disease symptoms by *E. coli* infection followed by antibiotic treatment in the EAE model. The overall mortality of the *E. coli* infection with the antibiotic treatment protocol employed was approximately 20% in our study. Leaving the infection untreated would have resulted in an unacceptably high mortality. The temporary systemic infection followed by antibiotic treatment mimics the situation in well-treated acutely ill patients. However, the effect of a chronic *E. coli* on the course of EAE remains to be determined.

In conclusion, in our model of MOG-EAE neither the clinical course nor the neurodegeneration was aggravated by systemic infection with *E. coli*. Our results suggest that the effect of moderately severe adequately treated systemic infections by *E. coli* on MS is less pronounced than suspected. Given the lack of efficacy of clinical and experimental trials based on targets identified in LPS studies [12, 41], our data confirm the complexity of systemic infections with viable bacteria compared to LPS-induced inflammation and underline the necessity of adequate infection models for testing the role of systemic infection in the pathogenesis of MS.

## Abbreviations

CNS: central nervous system
CFU: colony forming units
EAE: experimental autoimmune encephalomyelitis
LPS: lipopolysaccharide
UTI: urinary tract infections
MOG: myelin oligodendrocyte glycoprotein
MS: multiple sclerosis
ON: optic nerve
Pi: post immunization
RGCs: retinal ganglion cells
